# Associations of plasma MMP-9 and MPO with functional outcome in moderate to severe acute ischemic stroke

**DOI:** 10.1038/s41598-026-61410-z

**Published:** 2026-07-10

**Authors:** Vivian Vogt, Christoph Vollmuth, Guido Stoll, Felipe A. Montellano, Peter U. Heuschmann, Alexander M. Kollikowski, Mirko Pham, Karl Georg Haeusler, Hermann Neugebauer, Michael K. Schuhmann

**Affiliations:** 1 Department of Neurology, University Hospital Würzburg, Würzburg, Germany; 2 Institute of Experimental Biomedicine, University Hospital Würzburg, Würzburg, Germany; 3 Institute for Clinical Epidemiology and Biometry, University of Würzburg, Würzburg, Germany; 4 Institute for Medical Data Science, University of Würzburg, Würzburg, Germany; 5 University Hospital Würzburg, Clinical Trial Center, Würzburg, Germany; 6 Department of Neuroradiology, University Hospital Würzburg, Würzburg, Germany; 7 Department of Neurology, University Hospital Ulm, Ulm, Germany; 8 Department of Neurology, University Hospital Würzburg, Josef-Schneider-Str. 11, 97080 Würzburg, Germany

**Keywords:** Myeloperoxidase, Matrix metalloproteinase-9, Stroke, Biomarker, Biomarkers, Diseases, Medical research, Neurology, Neuroscience

## Abstract

Inflammation plays a pivotal role in acute ischemic stroke (AIS), with neutrophil granulocytes acting as major contributors to the post-stroke immune response. Upon degranulation, neutrophils release matrix metalloproteinase-9 (MMP-9) and myeloperoxidase (MPO), both of which may influence functional outcomes following AIS. The aim of this study was to investigate the association between systemic levels of MMP-9 and MPO, functional outcome, and neutrophil counts in patients with moderate to severe AIS, with a focus on their pathophysiological relevance rather than standalone prognostic performance. This prospective single-center cohort study included patients with moderate to severe AIS in the anterior circulation (NIHSS score ≥ 6 points and /or indication for mechanical recanalization). Venous blood was sampled in order to assess plasma concentrations of MMP-9 via zymography as well as MPO plasma levels via ELISA. Furthermore, differential blood count was determined simultaneously to venous biomarker sampling. Poor outcome was defined as mRS score ≥ 3. Uni- and multivariable regression analyses were performed. Between 07/2020 and 09/2022 a total of 279 patients with blood samples taken up to 48 h after symptom onset with a median NIHSS score of 13 and a median age of 79 were included. Of these patients, 81.0% underwent mechanical recanalization and 42.7% received systemic thrombolytic therapy. Systemic MMP-9 and MPO plasma levels were significantly higher in patients with poor functional outcome compared to patients with good functional outcome at 3-months (MMP-9: 359.1 vs. 303.5 ng/ml, *p* = 0.0006; MPO: 27.0 vs. 19.2 ng/ml, *p* = 0.0010). Both biomarkers were associated with a poor outcome in unadjusted analyses [MMP-9: unadjusted OR = 15.63 (95% CI: 3.54-80.69), *p* = 0.0005; MPO: unadjusted OR = 4.02 (95% CI: 1.84-9.21), *p* = 0.0007]. In addition, MMP-9 and MPO concentrations correlated with neutrophil counts (MMP-9: *r* = 0.39, *p* < 0.0001; MPO: *r* = 0.31, *p* < 0.0001). In conclusion, systemic MMP-9 and MPO plasma concentrations are associated with functional outcome in patients with moderate to severe AIS and reflect neutrophil-driven inflammatory processes. Their value appears to lie in complementing established clinical predictors rather than serving as independent prognostic markers.

**Supplementary Information:**

The online version contains supplementary material available at 10.1038/s41598-026-61410-z.

## Introduction

With around seven million deaths worldwide, stroke is the second leading cause of death among non-communicable diseases. Furthermore, it is the third leading cause of death and disability, resulting in an annual global cost of around 890 billion dollars^1^. In light of this global burden, a deeper understanding of stroke pathophysiology is needed in order to develop and improve treatment options, and reliable biomarkers for prognostication need to be identified. One promising avenue for both lies in the investigation of immune and vascular responses that occur immediately after vessel occlusion. Emerging evidence indicates significant intravascular inflammatory responses in cases of large vessel occlusion stroke, predominantly characterized by neutrophil recruitment and platelet activation, which contribute to early tissue injury^2–4^. Among the molecular players involved, matrix metalloproteinase-9 (MMP-9) has emerged as a mediator of tissue damage^5^. Recent evidence has shown that MMP-9 is locally upregulated in neutrophils during acute ischemia and was indicative of parenchymal hematomas, severe disability or death^6^. This process is, in part, driven by neutrophil degranulation, which leads to the release of gelatinase granules containing MMP-9^7^. MMP-9 belongs to a multigene family with zinc-binding motifs^8^ and its enzymatic activity has been implicated in the breakdown of the blood-brain barrier (BBB) following ischemic stroke^7,9,10^. In addition to MMP-9, another molecule released during degranulation of neutrophils is myeloperoxidase (MPO), one of the most abundant proteins in neutrophils^11^. MPO can directly damage the vascular wall by oxidizing its components^12^. Given their involvement in inflammatory cascades during acute ischemic stroke (AIS), the present study investigates MMP-9 and MPO as circulating markers of neutrophil activation. Specifically, we examine the association between systemic plasma levels of MMP-9 and MPO and functional outcomes at 3 months in patients with moderate to severe AIS, with a particular focus on their pathophysiological relevance within neutrophil-driven inflammatory responses.

## Results

### Baseline characteristics

**Fig. 1:**
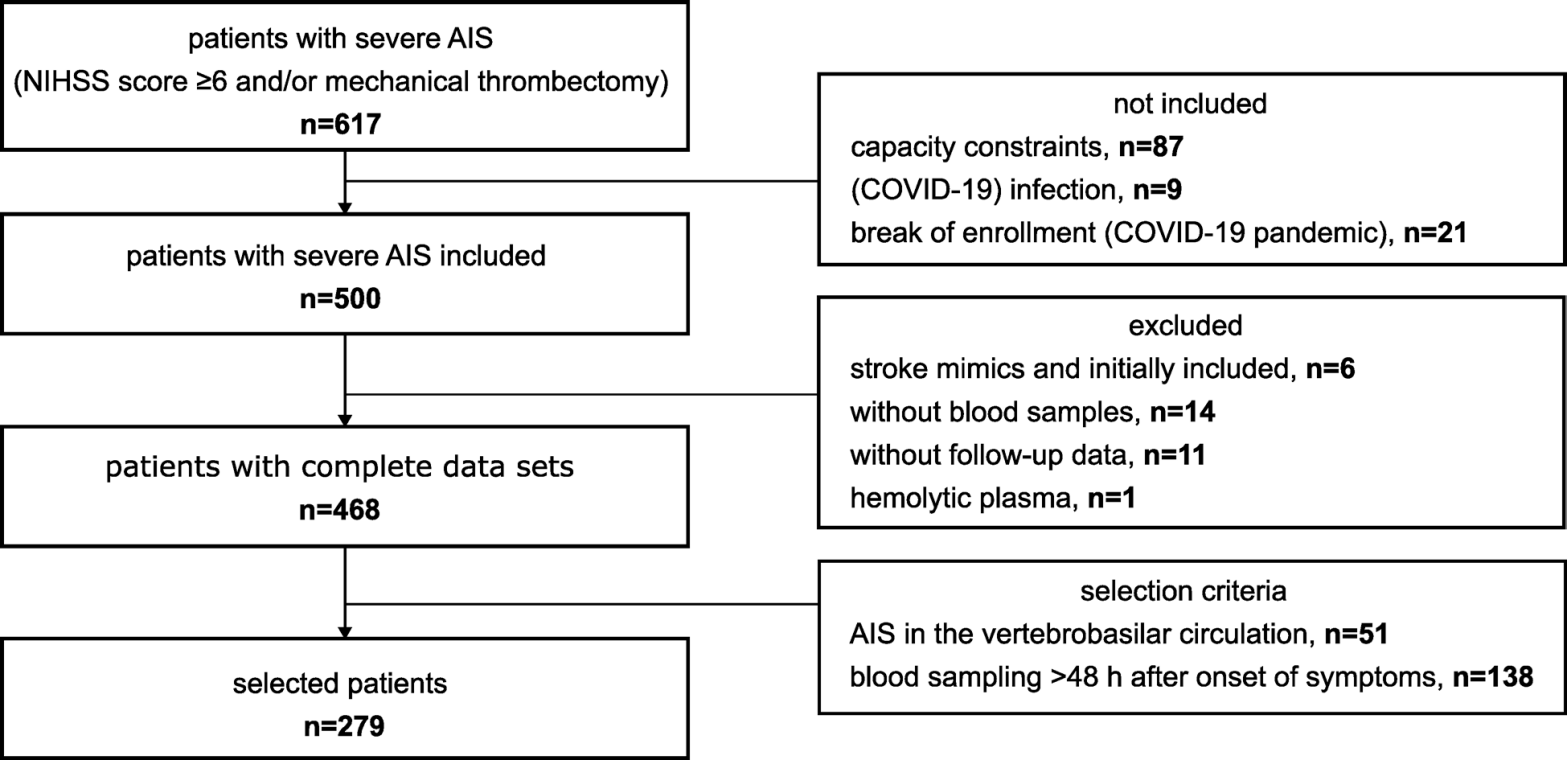
Final Cohort Selection Process.

Between June 2020 and September 2022, 617 patients with moderate to severe AIS, who were initially eligible for the study, were admitted to the University Hospital Würzburg in Germany. Due to capacity constraints, 87 patients were not included. Furthermore, the COVID-19 pandemic caused a halt in enrollment between December 2020 and January 2021, resulting in 21 stroke patients who could not be included in this study. Further 9 patients presenting with a COVID-19 infection were not included. Initially 500 patients were included, but no blood samples could be obtained from 14 patients and the plasma of 1 patient was hemolytic. A further 11 patients did not participate in follow-up and 6 patients with stroke mimics were excluded from analysis. Because of selection criteria another 51 patients experiencing AIS in the vertebrobasilar circulation were excluded, along with 138 patients whose blood was sampled more than 48 h after stroke onset. Data from the 138 patients with blood sampling beyond 48 h after stroke onset were analyzed separately and are provided in the Supplementary Materials. Overall, this comprises a cohort of 279 patients (Fig. 1). An overview of included and excluded patients with respect to age, sex and NIHSS score on admission is provided in table S1. As no patients sampled in June met the inclusion criteria for the present analysis the cohort comprises patients enrolled between July 2020 and September 2022.

**Fig. 2:**
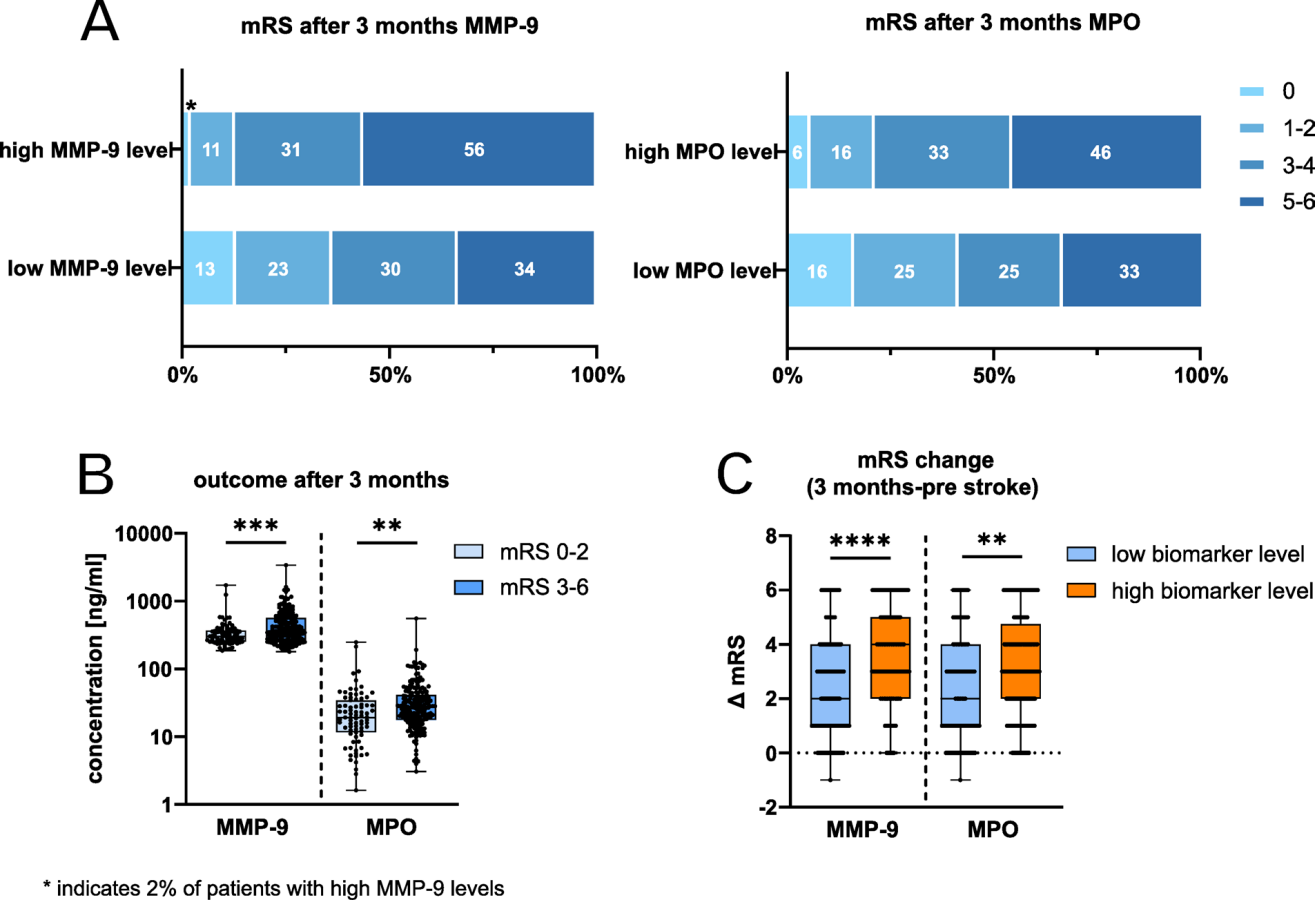
Low MMP-9 and MPO Plasma Levels Yield Better Functional Outcome. Functional outcome in terms of **(A)** mRS after 3 months for patients with high or low biomarker levels (* on the upper bar indicates 2% of patients with high MMP-9 levels and a mRS score of 0), **(B)** mRS after 3 months categorized into two groups (mRS 0-2 = good outcome and mRS 3-6 = poor outcome) and **(C)** Δ mRS (mRS after 3 months - mRS before stroke) in patients with low or high biomarker levels. mRS score is indicated in the legend, numbers in the bar graph show patients in %. Cut-off values of 410.0 ng/ml for MMP-9 and 19.4 ng/ml for MPO were defined by Youden Index. **p-value < 0.01; ***p-value < 0.001; *n* = 279.

A total of 279 patients [*n* = 147 (52.7%) female, median age 79 (IQR: 67-84) years, median NIHSS score on admission 13 (IQR: 9-17) points, median ASPECTS on admission 8 (IQR: 6-9)] were included in this study (Table 1). Systemic thrombolysis was performed in 119 patients (42.7%), while 226 (81.0%) received mechanical thrombectomy. The distribution of functional outcome according to high and low biomarker levels is shown in (Fig. 2A). In this cohort, 79 (28.3%) patients had a good functional outcome after 3 months, while 200 (71.7%) had a poor functional outcome, including 102 (36.6%) patients who died. Regarding vascular risk factors, patients with diabetes mellitus showed a significantly higher ratio for poor outcome (*p* = 0.0301). No significant differences were shown for suspected etiology of stroke and prior medication with oral platelet aggregation inhibitors. Baseline data of patients with blood obtained over 48 h compared to patients with blood sampled up to 48 h after stroke onset is provided in table S2.

**Table 1:** Baseline Data.

Baseline data	Total (*n* = 279)	good outcome after 3 months (*n* = 79)	poor outcome after 3 months (*n* = 200)	*p*-value^b^
Age, median [IQR]	79 [67-84]	72 [62-81]	80 [69-85]	**< 0.0001**
Sex, female, n (%)	147 (52.7)	26 (32.9)	121 (60.5)	**< 0.0001**
Pre-stroke mRS 0-2, n (%)	229 (82.1)	79 (100)	150 (75.0)	**< 0.0001**
Vascular risk factors, n (%)				
Hypertension	178 (63.8)	45 (57.0)	133 (66.5)	0.17
Diabetes mellitus	55 (19.7)	9 (11.4)	46 (23.0)	**0.0301**
Heart failure	36 (12.9)	6 (7.6)	30 (15.0)	0.11
Atrial fibrillation	125 (44.8)	34 (43.0)	91 (45.5)	0.79
Stroke severity^a^, median [IQR]				
NIHSS on admission	13 [9-17]	10 [7-16]	14 [10-18]	**< 0.0001**
NIHSS at 24 h after admission	12 [4-20]	3 [1-6]	15 [9-26]	**< 0.0001**
NIHSS at 48 h after admission	10 [3-18]	2 [1-4]	14 [7-22]	**< 0.0001**
NIHSS at 72 h after admission	7 [216]	2 [0-3]	13 [6-20]	**< 0.0001**
NIHSS at discharge	4 [1-10]	1 [0-2.25]	8 [3.25-13.0]	**< 0.0001**
Suspected etiology, n (%)				
Large artery atherosclerosis	48 (17.2)	17 (21.5)	31 (15.5)	0.29
Cardioembolism	127 (45.5)	35 (44.3)	92 (46.0)	0.79
Cryptogenic	88 (31.5)	24 (30.4)	64 (32.0)	0.89
Other causes	8 (2.9)	2 (2.5)	6 (3.0)	> 0.99
Concurrent causes	8 (2.9)	1 (1.3)	7 (3.5)	0.45
Brain imaging data Cerebral artery occluded, n (%)				
Carotis-T	28 (10.0)	2 (2.5)	26 (13.0)	**0.0133**
M1	168 (60.2)	43 (54.4)	125 (62.5)	0.22
M2	60 (21.5)	23 (29.1)	37 (18.5)	0.05
ASPECTS on admission, median [IQR]	8 [6-9]	9 [8-9]	7 [6-8]	**< 0.0001**
Acute stroke treatment, n (%)				
Systemic thrombolysis	119 (42.7)	42 (53.2)	77 (38.5)	**0.0315**
Symptom onset to systemic thrombolysis, hours, median [IQR]	1.7 [1.2-2.4]	1.5 [1.2-2.3]	1.7 [1.3-2.6]	0.44
Mechanical thrombectomy	226 (81.0)	58 (73.4)	168 (84.0)	0.0613
Symptom onset to femoral artery puncture for mechanical recanalization, hours, median [IQR]	4.2 [2.7-8.4]	3.5 [2.2-6.2]	4.4 [3.1-11.4]	**0.0180**
TICI ≥2b	197 (87.2)	57 (98.3)	140 (83.3)	**0.0054**
Prior medication, n (%)				
Antiplatelet	123 (44.1)	40 (50.6)	83 (41.5)	0.18
Acetylsalicylic acid	116 (41.6)	39 (49.4)	77 (38.5)	0.11
Clopidogrel	15 (5.4)	3 (3.8)	12 (6.0)	0.57
Symptom onset to blood sample, hours, median [IQR]	26.3 [20.0-36.5]	25.0 [19.0-35.0]	27.4 [20.0-37.5]	0.18
Biomarker data, median [IQR]				
MMP-9 (ng/ml)	341.9 [263.2-490.3]	303.5 [252.7-371.0]	359.1 [269.0-568.9]	**0.0006**
With antiplatelet agents *n* = 123	339.6 [263.2-517.7]	304.6 [247.2-362.8]	357.1 [272.0-585.9]	**0.0038**
Without antiplatelet agents *n* = 156	343.2 [263.3-490.1]	289.2 [257.6-396.5]	361.1 [265.0-521.3]	**0.0455**
MPO (ng/ml)	24.6 [15.6-38.5]	19.2 [11.7-34.6]	27.0 [17.7-41.5]	**0.0010**
With antiplatelet agents *n* = 123	23.2 [15.6-38.4]	19.3 [13.0-29.1]	26.0 [17.3-45.4]	**0.0434**
Without antiplatelet agents *n* = 156	25.6 [15.5-38.6]	19.1 [8.4-37.9]	27.3 [17.9-39.8]	**0.0103**

^a^Deceased patients excluded.

^b^Patients with good functional outcome vs. poor outcome.

### Univariable analysis of systemic plasma levels of MMP-9 and MPO at baseline and functional outcome

Plasma levels of MMP-9 and MPO were significantly higher in patients with poor outcome, if compared to patients with good outcome at 3 months [median MMP-9: 359.1 ng/ml (IQR: 269.0-568.9) vs. 303.5 ng/ml (IQR: 252.7-371.0), *p* = 0.0006; median MPO: 27.0 ng/ml (IQR: 17.7-41.5) vs. 19.2 ng/ml (IQR: 11.7-34.6), *p* = 0.0010] (Fig. 2B). Respective cut-off values for biomarker concentrations were assessed by Youden Index (MMP-9: 410.0 ng/ml; MPO: 19.4 ng/ml). To account for high pre-stroke mRS scores, the change between pre-stroke mRS and 3-month mRS score was calculated and plotted for low and high biomarker levels (Fig. 2C). Patients with high peripheral MMP-9 plasma concentrations after AIS [median mRS score: 4.00 (IQR: 2.00-5.00) vs. 2.00 (IQR: 1.00-4.00), *p* < 0.0001], as well as patients with high MPO concentrations after AIS [median mRS score: 3.00 (IQR: 2.00-4.75) vs. 2.00 (IQR: 1.00-4.00), *p* = 0.0033] showed a significant increase in delta mRS score after 3 months.

### Stroke severity and etiology

**Fig. 3:**
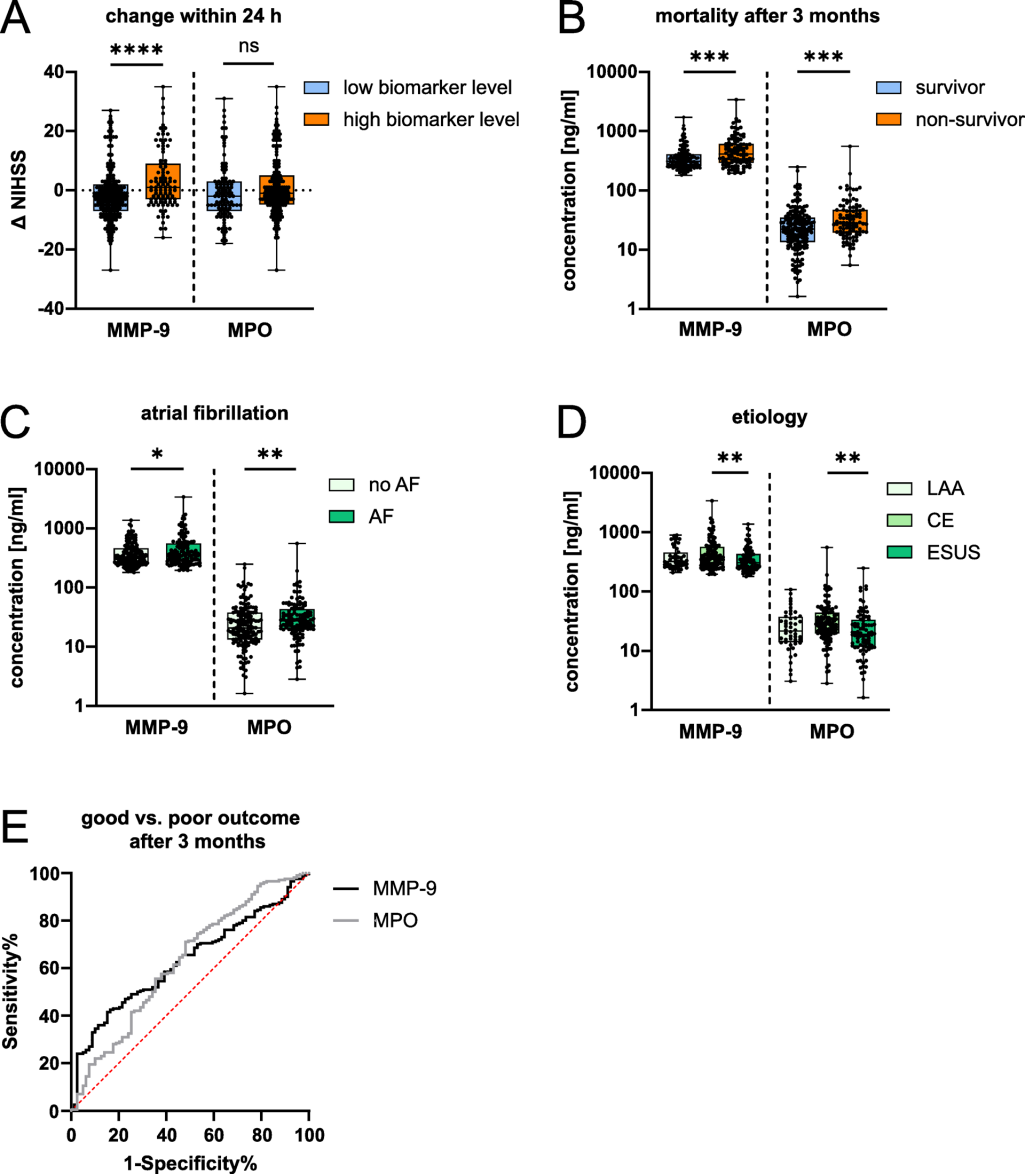
Prognostic Relevance and Etiological Discrimination Potential of MMP-9 and MPO. (**A**) Δ NIHSS (NIHSS score after 24 h - NIHSS score on admission) in patients with biomarker levels according to cut-off value (MMP-9: 410.0 ng/ml; MPO: 19.4 ng/ml). (**B**) Biomarker levels and survival after 3 months. (**C**) Biomarker levels and atrial fibrillation (AF). (**D**) Etiology of stroke for MMP-9 and MPO levels (LAA: large artery atherosclerosis, CE: cardioembolism, ESUS: embolic stroke of undetermined source) (*n* = 263). (**E**) ROC analysis of MMP-9 (AUC = 0.63, 95% CI: 0.56-0.70) and MPO (AUC = 0.63, 95% CI: 0.55-0.70) for functional outcome after 3 months (mRS 0-2 vs. 3-6). *p-value < 0.05; **p-value < 0.01; ***p-value < 0.001; *n* = 279.

Short-term functional outcome was assessed using the change in NIHSS score from admission to 24 h. Biomarker levels were again divided into two groups with either high or low levels, according to the previously defined cut-off. A significant difference was observed for plasma levels of MMP-9 (*p* < 0.0001), but not for MPO (*p* = 0.08). In the 95 patients with high MMP-9 concentrations, the median NIHSS score increased by one point (IQR: -3-9) within 24 h after admission, while the 184 patients with low MMP-9 concentrations showed a decrease in the NIHSS score by two points (IQR: -7-2). The NIHSS score on admission decreased by one point (IQR: -4.75-5.0) in the 180 patients with high MPO levels, whereas it was reduced by two points (IQR: -7.0-3.0) in the 99 patients with low MPO concentrations (Fig. 3A). Next, we found significantly higher MMP-9 plasma concentrations in patients, who died within 3 months, compared to survivors [median: 413.6 ng/ml (IQR: 292.5-611.0) vs. 310.5 ng/ml (IQR: 256.5-409.4), *p* = 0.0001]. This was also shown for MPO plasma levels (median: 28.2 ng/ml [IQR: 19.4-47.1] vs. 22.2 ng/ml [IQR: 13.5-34.9], *p* = 0.0007) (Fig. 3B). To account for stroke severity, patients were categorized according to the Heidelberg Bleeding Classification, based on the severity of intracranial bleeding. No significantly higher biomarker levels were observed in patients with more severe bleeding (Fig. S1). If patients had known atrial fibrillation (AF) prior to the index stroke, higher levels of both biomarkers were detected in venous blood samples after AIS [median MMP-9: 360.0 ng/ml (IQR: 277.9-555.1) vs. 329.6 ng/ml (IQR: 254.7-461.9), *p* = 0.0377; median MPO: 27.5 ng/ml (IQR: 19.7-43.1) vs. 20.8 ng/ml (13.3-38.0), *p* = 0.0049] (Fig. 3C). Regarding etiology of stroke, patients with cardioembolism (CE) had higher systemic biomarker levels compared to patients with embolic stroke of undetermined source (ESUS) [median MMP-9: 364.6 ng/ml (IQR: 280.4-567.4) vs. 308.8 ng/ml (IQR: 247.4-431.7), *p* = 0.0084; median MPO: 27.5 ng/ml (IQR: 19.7-44.2) vs. 19.0 ng/ml (IQR: 11.8-33.6), *p* = 0.0012] (Fig. 3D). The data corresponding to blood samples collected beyond 48 h after stroke onset are presented in the supplementary material (Fig. S2).

### Univariable and multivariable regression analysis of systemic plasma levels of MMP-9 and MPO

The univariable ROC-curve predicting 3-month functional outcome (mRS 0-2 vs. 3-6) showed an area under the curve (AUC) of 0.63 (95% CI: 0.56-0.70) for MMP-9 and an AUC of 0.63 (95% CI: 0.55-0.70) for MPO (Fig. 3E). The AUC was 0.62 (95% CI: 0.55-0.69) when both biomarkers were combined yielding no higher level of prediction. A model combining both biomarkers, admission NIHSS score, baseline neutrophil count, and patient age yielded an AUC of 0.75 (95% CI: 0.69-0.81) (Table 2). DeLong’s test showed no significant incremental contribution of MMP-9 and MPO to models including age and admission NIHSS score (Tab. S3). Additional ROC analyses for different combinations of the mentioned variables are provided in Table S4. To gain further insights into the predictive value of MMP-9 and MPO, unadjusted and adjusted regression analyses for mortality and functional outcome after 3 months were performed. Plasma levels of MMP-9 were associated with poor functional outcome [OR: 15.63 (95% CI: 3.54-80.69), *p* = 0.0005] and mortality [OR: 13.93 (95% CI: 4.15-50.21), *p* < 0.0001] in unadjusted analysis (Tab. S5). The association remained significant after adjusting for established predictors [patients´ age, NIHSS score on admission, ASPECTS on admission, recanalization therapy (yes/no)] [OR: 12.11 (95% CI: 2.16-80.51)] for functional outcome (Table 3) as well as for mortality [OR: 9.66 (95% CI: 2.36-42.83)]. After adjustment for the NIHSS score 24 h after admission, the association with mortality remained [OR: 6.60 (95% CI: 1.43-32.46)] (Table 4), whereas no significant association with functional outcome was observed [OR: 5.04 (95% CI: 0.76-38.52)] (Table 3). Following additional adjustment for diabetes mellitus and pre-stroke mRS, and accounting for NIHSS score at 24 h after admission, the association of MMP-9 with functional outcome reached statistical significance [OR: 8.31 (95% CI: 1.10-71.55)] (Tab. S6). MPO plasma levels were linked to functional outcome [OR: 4.02 (95% CI: 1.84-9.21), *p = 0.0007*] and mortality [OR: 4.01 (95% CI: 1.91-8.84), *p* = 0.0004] at 3 months in the unadjusted analysis. After adjustment for the aforementioned established predictors, MPO levels were associated with 3-month outcome [OR: 2.70 (95% CI: 1.17-6.52)] and mortality [OR: 3.34 (95% CI: 1.42-8.30)]. When adjusting not for the NIHSS score on admission but for the NIHSS score 24 h after admission MPO remained associated with mortality [OR: 3.21 (95% CI: 1.24-8.75)] but showed no significant association with functional outcome [OR: 2.36 (95% CI: 0.89-6.48)]. Adjustment for diabetes mellitus and pre-stroke mRS resulted in loss of the association of MPO with functional outcome after adjustment for NIHSS score on admission [OR: 2.12 (95% CI: 0.88-5.27)] (Tab. S6). For both biomarkers, associations with mortality remained significant after inclusion of these variables (Tab. S7). Additionally, logistic regression analyses stratified by sampling time are provided in the supplemental material (Tab. S8-S11).

**Table 2:** ROC Curves of Biomarker Concentrations, Age, NIHSS Score on Admission, Neutrophils, and Combined Variables (*n* = 279; in case of neutrophils or combined variables with neutrophils *n* = 273).

Variable	AUC [95% CI]
Age	0.66 [0.59-0.72]
NIHSS score on admission	0.68 [0.61-0.75]
Neutrophils	0.62 [0.54-0.69]
MMP-9 concentration	0.63 [0.56-0.70]
MPO concentration	0.63 [0.55-0.70]
MMP-9 + MPO concentration	0.62 [0.55-0.69]
NIHSS score on admission + MMP-9 concentration + MPO concentration	0.71 [0.64-0.78]
Age + NIHSS score on admission + neutrophils + MMP-9 concentration + MPO concentration	0.75 [0.69-0.81]

**Table 3:** Multivariable Analysis for Association of Systemic Biomarker Levels and 3-month Outcome.

Variable	Odds ratio* [95% CI]	Odds ratio** [95% CI]	Odds ratio* [95% CI]	Odds ratio** [95% CI]
Age	1.05 [1.02-1.08]	1.05 [1.02-1.09]	1.05 [1.02-1.08]	1.05 [1.02-1.09]
NIHSS score on admission	1.09 [1.03-1.16]	/	1.09 [1.03-1.15]	/
NIHSS score 24 h after admission	/	1.23 [1.15-1.32]	/	1.22 [1.15-1.32]
ASPECT Score	0.21 [0.10-0.41]	0.37 [0.17-0.81]	0.20 [0.10-0.40]	0.36 [0.16-0.78]
Recanalization therapy (yes/no)	0.52 [0.14-1.68]	0.60 [0.14-2.20]	0.67 [0.17-2.28]	0.74 [0.18-2.78]
MMP-9 log10	/	/	12.11 [2.16-80.51]	5.04 [0.76-38.52]
MPO log10	2.70 [1.17-6.52]	2.36 [0.89-6.48]	/	/

Odds ratios are reported for logarithmic increases of base 10.

* adjusted for age, NIHSS score on admission, ASPECTS dichotomized, recanalization therapy.

** adjusted for age, NIHSS score 24 h after admission, ASPECTS dichotomized, recanalization therapy.

**Table 4:** Multivariable Analysis for Association of Systemic Biomarker Levels and Mortality within 3 Months.

Variable	Odds ratio* [95% CI]	Odds ratio** [95% CI]	Odds ratio* [95% CI]	Odds ratio** [95% CI]
Age	1.08 [1.05-1.12]	1.09 [1.06-1.13]	1.08 [1.05-1.12]	1.09 [1.06-1.13]
NIHSS score on admission	1.08 [1.03-1.13]	/	1.07 [1.03-1.13]	/
NIHSS score 24 h after admission	/	1.11 [1.08-1.15]	/	1.11 [1.08-1.15]
ASPECT Score	0.37 [0.20-0.67]	0.57 [0.29-1.12]	0.38 [0.20-0.69]	0.58 [0.29-1.14]
Recanalization therapy (yes/no)	0.41 [0.13-1.31]	0.40 [0.12-1.31]	0.53 [0.17-1.60]	0.49 [0.15-1.59]
MMP-9 log10	/	/	9.66 [2.36-42.83]	6.60 [1.43-32.46]
MPO log10	3.34 [1.42-8.30]	3.21 [1.24-8.75]	/	/

Odds ratios are reported for logarithmic increases of base 10.

*adjusted for age, NIHSS score on admission, ASPECTS dichotomized, recanalization therapy.

**adjusted for age, NIHSS score 24 h after admission, ASPECTS dichotomized, recanalization therapy.

### Neutrophil granulocytes and neutrophil-to-lymphocyte ratio correlate with MMP-9 and MPO plasma concentrations

**Fig. 4:**
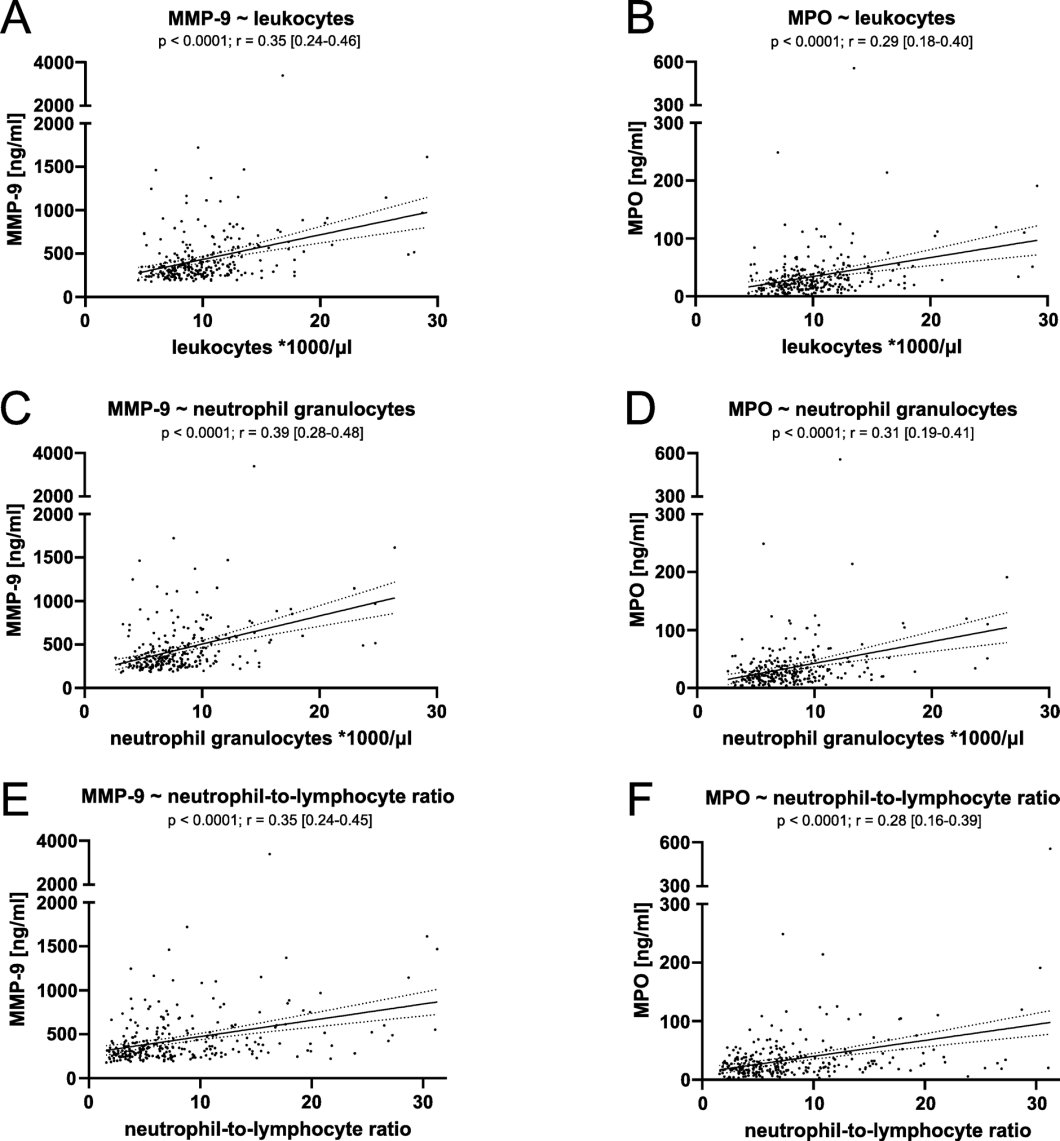
MMP-9 and MPO Concentrations in Stroke Patients Correlate with Neutrophil Granulocytes. Spearman’s correlations for MMP-9 and MPO and (**A**,** B**) leukocytes, (**C**,** D**) neutrophil granulocytes, (**E**,** F**) neutrophil-to-lymphocyte ratio (NLR); *n* = 273.

A differential blood count, simultaneously assessed to venous biomarker sampling, was performed to gain insight into possible correlations with biomarker levels. There were significant correlations between MMP-9 concentrations and leukocyte counts [*r* = 0.35 (95% CI: 0.24-0.46), *p* < 0.0001], the count of neutrophil granulocytes [*r* = 0.39 (95% CI: 0.28-0.48), *p* < 0.0001] and the neutrophil-to-lymphocyte ratio [*r* = 0.35 (95% CI: 0.24-0.45), *p* < 0.0001]. Plasma levels of MPO also significantly correlated with leukocytes [*r* = 0.29 (95% CI: 0.18-0.40), *p* < 0.0001], neutrophil granulocytes [*r* = 0.31 (95% CI: 0.19-0.41), *p* < 0.0001] and neutrophil-to-lymphocyte ratio [*r* = 0.28 (95% CI: 0.16-0.39), *p* < 0.0001] (Fig. 4). Correlations between MPO and MMP-9 with thrombocytes, lymphocytes and monocytes are presented in the supplementary material (Fig. S3). Furthermore, correlations of MMP-9 and MPO with different types of blood cells for blood sampled over 48 h after stroke onset are shown (Tab. S12).

## Discussion

As principal finding, we show that MMP-9 and MPO concentrations were significantly elevated in systemic plasma samples from AIS patients with poor functional outcome at 3 months after stroke. These findings primarily support the involvement of neutrophil-derived mediators in stroke-related inflammation rather than indicating strong independent prognostic utility. Notably, higher MMP-9 levels corresponded to poorer short-term functional outcome, reflected by changes in NIHSS scores within the first 24 h after admission. Elevated levels of both biomarkers were also associated with 3-month functional outcome and mortality.

These findings from our well-characterized cohort of patients with moderate to severe AIS are consistent with previous studies in patients with mild to moderate stroke severity. In one such cohort elevated MPO levels were associated with poorer outcomes, while active MMP-9 showed no predictive value^13^. In another study involving patients with mild AIS, increased serum MMP-9 levels, taken up to 24 h after admission, were associated with higher mortality and poor functional outcomes at 3 months^14^. However, the prognostic value of biomarkers may differ between patients with mild and those with more severe strokes^15^. As the aforementioned cohorts included only patients with mild stroke severity, the present study addresses a relevant gap by investigating MMP-9 and MPO in patients with moderate to severe AIS. This subgroup is underrepresented in biomarker research, yet highly relevant in clinical practice due to its higher risk of poor outcome and frequent eligibility for reperfusion therapy^15,16^. Studying this population enabled us to assess biomarker dynamics in the context of more extensive tissue injury and a more pronounced inflammatory response. Our findings further suggest that neutrophil-derived biomarkers may serve as indicators of the acute inflammatory response in more severe ischemic injury, which may be particularly relevant in the setting of reperfusion therapies.

A key finding of our study is the attenuation of the association between MMP-9 and MPO levels and functional outcome after adjustment for the NIHSS at 24 h. Given that the 24-hour NIHSS is a robust predictor of outcome^17^, this suggests that the relationship between these biomarkers and outcome is closely linked to early neurological evolution. In this context, MMP-9 and MPO likely reflect early infarct severity and dynamic processes, such as neutrophil activation and early tissue injury, rather than acting as fully independent prognostic markers. This interpretation is consistent with their known biological roles in BBB disruption and amplification of neuroinflammation. Thus, our findings support the view that these biomarkers provide insight into underlying pathophysiological mechanisms and therefore relevant biological processes of stroke progression, while their additional value for clinical risk prediction beyond established predictors appears limited in this cohort.

Both experimental and clinical studies have identified leukocytes, particularly neutrophils, as a major cellular source of MMP-9 in the context of stroke^18–21^. In the present study, we observed significant correlations between systemic MMP-9 plasma concentrations and neutrophils, further supporting the notion that neutrophils are a key contributor to circulating MMP-9 levels in AIS. Similarly, MPO levels also showed significant correlations with neutrophils, which is consistent with MPO being one of the most abundant proteins in neutrophils^11,22^. These findings are in line with the prospective monocentric cohort study, which reported that higher plasma concentrations of MMP-9 and MPO correlated with neutrophil counts in stroke patients^13^. In local arterial ischemic blood samples collected during routine endovascular thrombectomy MPO concentrations were not only found to correlate with locally assessed neutrophil counts but also with functional outcome at hospital discharge^3,23^, further supporting the clinical relevance of neutrophil-derived MPO in acute stroke. Beyond their association with systemic inflammation, both MMP-9 and MPO contribute to BBB disruption^12,24^, thereby facilitating immune cell infiltration into the brain parenchyma and amplifying neuroinflammation. In animal studies, both knock-out models and pharmacological inhibition approaches have been used to investigate the role of these proteins in cerebral ischemia. In particular, MMP-9 knock-out mice exhibited reduced BBB leakage. Moreover, infarct volumes measured 24 h after ischemia were significantly reduced compared to wild type mice^25^. Consistent with these findings, pharmacological inhibition of MMP-9 has been shown to enhance neuronal survival and synaptic integrity, reduce BBB disruption, and improve neurological outcomes^5,26^. Notably, MMP-9 levels were associated with an increased risk of hemorrhagic complications following endovascular thrombectomy in a cross-sectional proof-of-concept study^6^. Taken together, these findings support the concept that circulating levels of MMP-9 and MPO primarily reflect neutrophil activation and its downstream effects, including BBB disruption and amplification of neuroinflammation, rather than serving as independent prognostic markers.

## Limitations

While our results are encouraging, they must be interpreted in the context of the study’s strengths and limitations. A major strength of this prospectively designed study is its focus on patients with moderate to severe AIS, a population currently underrepresented in the biomarker literature.

Despite the relatively large sample size of 279 patients in this monocentric study, confirmation in larger, independent cohorts of patients with moderate to severe AIS is required to establish generalizability. The high proportion of poor outcomes reduced the effective sample size for modelling and resulted in imprecise estimates with wide confidence intervals. In combination with the modest discriminatory performance of the investigated biomarkers, we cannot exclude that the observed effect sizes are influenced by model instability or overfitting and should be interpreted with caution. Accordingly, the present analyses are best considered exploratory and hypothesis-generating. It should also be considered that blood samples were obtained at a median of 26 h (IQR: 20.0–36.5) after symptom onset, and MMP-9 and MPO levels were measured at only a single time point. This limits insight into the temporal dynamics of these biomarkers and precludes differentiation between early upstream and later downstream inflammatory responses. Future studies should include early and serial measurements to better characterize their temporal profile and pathophysiological relevance. Further, blood sampling was performed in all cases after completion of acute reperfusion therapy. No pre-treatment blood samples were available, leading to unknown baseline levels of MMP-9 and MPO. Those may have been influenced by pre-existing inflammatory conditions, causing higher biomarker levels already pre-stroke and therefore affecting interpretation. We also acknowledge that zymography is inherently semi-quantitative and may introduce measurement variability. Regarding statistical analysis it should be noted that although we included the acute ASPECTS as well as recanalization therapy in logistic regression analyses, no adjustment for infarct volume was conducted, possibly confounding results. Adjustment models were performed without correction for multiple testing, as the analyses were considered exploratory and hypothesis-generating. Furthermore, we followed current statistical recommendations from the Prognosis Research Strategy (PROGRESS) Partnership on prognostic factor research regarding the analysis of biomarkers as continuous variables^27^. Thus, we report odds ratios for logarithmic increases of the biomarkers’ values. Importantly, this approach may limit the direct clinical interpretability of the studied biomarkers. However, since individual predictors are rarely sufficient for reliable prognostic estimation^28^, ideal integration of prognostic biomarker measurement into clinical practice include its integration into multivariable prognostic models^29^, thus enabling a balance between robust statistical methodology and meaningful clinical interpretation^15^.

## Conclusion

In conclusion, plasma concentrations of the neutrophil-derived proteins MMP-9 and MPO were associated with functional outcome and mortality in patients with moderate to severe AIS. While their independent prognostic value beyond established clinical predictors is limited, their correlation with neutrophil counts highlights their relevance as markers of neutrophil-driven inflammation in stroke pathophysiology.

## Methods

Patients with moderate to severe AIS were prospectively studied in this single-center observational cohort study. Patients presenting an admission NIHSS score ≥ 6 points and/or undergoing mechanical recanalization due to occlusion of a large intracranial vessel in the anterior circulation (regardless of their NIHSS score) were recruited. We restricted the analysis to anterior circulation strokes as stroke severity is reflected best by the NIHSS in this region while its application to the posterior circulation might lead to misclassification of clinical severity. Venous blood samples were taken up to 48 h after symptom onset. Patients had to be at least 18 years old and needed a sufficient proficiency in German to be included. Furthermore, participation in interventional trials within the past 3 months that potentially affect platelet function or stroke outcomes led to exclusion.

### Ethics approval and consent to participate

This study is in accordance with the Declaration of Helsinki and its later amendments. All participants or their legal representatives gave written informed consent. The local Ethics Committee of the University of Würzburg approved this study (reference n. 05/20-am), which was registered under DRKS00022064.

### Central clinical database

To ensure comprehensive documentation of demographic and clinical variables data were assessed in a centralized database. NIHSS scores were determined upon admission, at 24 h, 48 h and 72 h, as well as at hospital discharge. Experienced neuroradiologists used the Alberta Stroke Program Early CT Score (ASPECTS) to evaluate early infarct signs in non-contrast CT scans on admission and independently classified the expanded Treatment In Cerebral Ischemia (eTICI) for patients who received mechanical thrombectomy. The Trial of ORG 10172 in Acute Stroke Treatment (TOAST) criteria served for categorizing the etiology of ischemic stroke based on the information available at discharge. Furthermore, patient interviews and chart reviews were used to determine comorbidities and cardiovascular risk factors. Grade of disability was evaluated with the modified Rankin Scale (mRS) before admission. For functional outcome the mRS after 3 months (± 14 days) post AIS was acquired via telephone interview. A mRS score of 0-2 defined a good functional outcome.

### Blood sampling and analysis

The morning after study enrollment blood samples were taken from the antecubital vein into a Citrat 4.3 ml S-Monovette (Sarstedt, Germany). In all cases, sampling was performed after completion of acute reperfusion therapy (intravenous thrombolysis and/or mechanical thrombectomy). The sample was centrifuged at 2500 x g for 10 min and 360 µl were aliquoted into 1.0 storage tubes. Samples were stored at -80 °C. MPO plasma concentrations were quantified by ELISA (Human Myeloperoxidase Instant ELISA™ Kit, invitrogen, BMS2038INST). For quantification a four parameter logistic regression (4PL) was used in GraphPad Prism 10.3.1 (GraphPad Software, La Jolla, USA). MMP-9 plasma levels were assessed by gelatine zymography as described previously^30^. For SDS PAGE separation 1 µl of plasma was used. As protein standard recombinant human MMP-9 protein (SAE0077, Sigma-Aldrich, Taufkirchen, Germany) was diluted in sample buffer and loaded onto the gels in concentrations of 5, 1 and 0.2 ng for semiquantitative analysis. A BioRad ChemiDoc™ Imaging System (Hercules, California, USA) was used to take images. For each gel the last image taken before overexposure was analyzed with ImageJ 1.54f (National Institutes of Health, USA). For quantification a semilog line was used in Graphpad. Intra- and inter-assay variability, calculated as coefficients of variation (CVs) using pooled plasma samples from healthy donors, were 3.6% and 15.5%, respectively.

### Statistical analysis

For statistical analysis, R 4.4.2 (R Foundation, Vienna, Austria) and GraphPad Prism 10.3.1 (GraphPad Software, La Jolla, USA) were used. Categorical variables are shown as patient numbers with percentages (%), while continuous variables were reported as median with interquartile range (IQR). D’Agostino & Pearson test was used to test Gaussian distribution. Comparison of two groups was performed with the two-tailed Mann-Whitney U test. The Spearman’s coefficient was used to calculate correlations between biomarker levels. For comparison of three or more variables the Kruskal-Wallis test with Dunn’s multiple comparisons test was used. Multicollinearity was assessed by examination of the correlation, and no variables demonstrated associations ≥ 0.8. The subtraction of the NIHSS score on admission from the NIHSS score 24 after admission comprised the change in NIHSS score. Logistic regression analysis to assess associations of blood-based biomarkers with poor outcome and/or mortality was performed with logarithmically transformed biomarker levels due to skewed distribution. No adjustment for multiple comparisons was performed, and results should be interpreted as exploratory. We report odds ratios (OR) with 95% confidence intervals (CI) showing changes per log-unit. Adjustment was conducted for variables that have been identified as the most important covariates [(age, NIHSS score on admission respectively NIHSS score 24 h after admission, ASPECT score on admission and recanalization therapy (yes/no)]^16,31^. Areas under the curve (AUC) served as indication for prognostic values. Cut-off values for systemic biomarker concentrations were defined by Youden Index. To assess the diagnostic value for disability, uni- and multivariable receiver-operating-characteristic (ROC) analysis were performed with the variables biomarker concentrations, NIHSS on admission, age and neutrophil count. AUCs for multivariable ROC analyses were calculated using R 4.4.2, and 95% confidence intervals were estimated via bootstrapping with 1000 resamples. Statistical significance was defined by p-values < 0.05. Tests were performed two-tailed. Figures were created with Inkscape 1.3.2.

## Supplementary Information

Below is the link to the electronic supplementary material.

Supplementary Material 1

## Data Availability

Data will be made available upon reasonable request.
