# Supplementary material for: Associations of plasma MMP-9 and MPO with functional outcome in moderate to severe acute ischemic stroke
Source: Sci Rep. 2026 Jul 10;16:21612. doi: 10.1038/s41598-026-61410-z (PMC13354770; doi:10.1038/s41598-026-61410-z)
Supplement: Supplementary file 1 — Supplementary Material 1 [file 41598_2026_61410_MOESM1_ESM.docx]

**Tab. S1** Baseline Age, Sex and NIHSS for Included and Excluded Patients.

| **Variable** | **Patients with blood samples taken within 48 h after stroke onset (n=279)**  **(included)** | **Patients with blood samples taken over 48 h after stroke onset (n=138)**  **(excluded)** | **Patients with strokes occurring in the vertebrobasilar circulation (n=51)**  **(excluded)** |
| --- | --- | --- | --- |
| Age | 79 [67-84] | 78 [68-84] | 76 [63-83] |
| Sex | 147 (52.7) | 77 (55.8) | 20 (39.2) |
| NIHSS score on admission | 13 [9-17] | 13 [9-16] | 12 [8-29] |

**Tab. S2** Comparison of Manuscript Cohort with Patients with Blood Samples over 48 h.

| **Baseline data** | **Blood sampling ≤ 48 h**  **(n=279)** | **Blood sampling > 48 h (n=138)** | **p-value** |
| --- | --- | --- | --- |
| Age, median [IQR] | 79 [67-84] | 78 [68-84] | 0.9068 |
| Sex, female, n (%) | 147 (52.7) | 77 (55.8) | 0.5499 |
| Pre-stroke mRS 0-2, n (%) | 229 (82.1) | 117 (84.8) | 0.4904 |
| **Vascular risk factors, n (%)** |  |  |  |
| Hypertension | 178 (63.8) | 96 (69.6) | 0.2439 |
| Diabetes mellitus | 55 (19.7) | 28 (20.3) | 0.8903 |
| Heart failure | 36 (12.9) | 20 (14.5) | 0.6551 |
| Atrial fibrillation | 125 (44.8) | 69 (50.0) | 0.3176 |
| **Stroke severity^a^, median [IQR]** |  |  |  |
| NIHSS on admission | 13 [9-17] | 13 [9-16] | 0.4079 |
| NIHSS at 24 h after admission | 12 [4-20] | 13 [5-19.25] | 0.5087 |
| NIHSS at 48 h after admission | 10 [3-18] | 11 [4-18] | 0.2042 |
| NIHSS at 72 h after admission | 7 [2-16] | 10 [4-16] | 0.1030 |
| NIHSS at discharge | 4 [1-10] | 5 [2-12] | **0.0150** |
| **Suspected etiology, n (%)** |  |  |  |
| Large artery atherosclerosis | 48 (17.2) | 19 (13.8) | 0.3695 |
| Cardioembolism | 127 (45.5) | 68 (49.3) | 0.4704 |
| Cryptogenic | 88 (31.5) | 40 (29.0) | 0.5952 |
| Other causes | 8 (2.9) | 6 (4.3) | 0.4310 |
| Concurrent causes | 8 (2.9) | 5 (3.6) | 0.6774 |
| **Brain imaging data**  Cerebral artery occluded, n (%) |  |  |  |
| Carotis-T | 28 (10.0) | 15 (10.9) | 0.7931 |
| M1 | 168 (60.2) | 70 (50.7) | 0.0658 |
| M2 | 60 (21.5) | 38 (27.5) | 0.1724 |
| ASPECTS on admission, median [IQR] | 8 [6-9] | 7 [6-9] | 0.5472 |
| **Acute stroke treatment, n (%)** |  |  |  |
| Systemic thrombolysis | 119 (42.7) | 48 (34.8) | 0.1233 |
| Symptom onset to systemic Thrombolysis, hours, median [IQR] | 1.7 [1.2-2.4] | 1.5 [1.3-3.0] | 0.9979 |
| Mechanical thrombectomy | 226 (81.0) | 97 (70.3) | **0.0139** |
| Symptom onset to femoral Artery puncture for mechanical recanalization, hours, median [IQR] | 4.2 [2.7-8.4] | 4.3 [2.7-11.3] | 0.5359 |
| TICI ≥2b | 197 (87.2) | 85 (61.6) | 0.0645 |
| Symptom onset to blood  sample, hours, median [IQR] | 26.3 [20.0-36.5] | 74.8 [62.3-94.0] | **<0.0001** |
| **Biomarker data, median [IQR]** |  |  |  |
| MMP-9 (ng/ml) | 341.9 [263.2-490.3] | 350.9 [259.6-524.3] | 0.7569 |
| MPO (ng/ml) | 24.6 [15.6-38.5] | 32.5 [20.5-50.1] | **0.0003** |

^a^Deceased patients excluded.

**Tab. S3** DeLong's Tests for ROC Curves of Age, NIHSS Score on Admission and Biomarker Concentrations.

| **AUROC** | | **p-value** |
| --- | --- | --- |
| Age + NIHSS score on admission | + MMP-9 concentration |  |
| 0.72 [0.66-0.78] | 0.63 [0.56-0.70] | 0.27 |
| Age + NIHSS score on admission | + MPO concentration |  |
| 0.72 [0.66-0.78] | 0.63 [0.55-0.70] | 0.68 |
| Age + NIHSS score on admission | + MMP-9 concentration + MPO concentration |  |
| 0.72 [0.66-0.78] | 0.62 [0.55-0.69] | 0.25 |

**Tab. S4** ROC Curves of Different Combinations of Biomarker Concentrations, Age, Neutrophils and NIHSS Score on Admission (n=279; in case of neutrophils or combined variables with neutrophils n=273).

| **Variable** | **AUC [95%CI]** |
| --- | --- |
| Age + neutrophils | 0.70 [0.64-0.77] |
| Age + MMP-9 concentration | 0.70 [0.64-0.77] |
| Age + MPO concentration | 0.67 [0.60-0.73] |
| Age + NIHSS score on admission | 0.72 [0.66-0.78] |
| NIHSS score on admission + MMP-9 concentration | 0.71 [0.64-0.78] |
| NIHSS score on admission + MPO concentration | 0.69 [0.62-0.75] |
| NIHSS score on admission + neutrophils | 0.70 [0.64-0.76] |
| Neutrophils + MMP-9 concentration | 0.64 [0.58-0.71] |
| Neutrophils + MPO concentration | 0.62 [0.55-0.68] |
| Age + NIHSS score on admission + neutrophils | 0.74 [0.68-0.80] |
| Age + NIHSS score on admission + MMP-9 concentration | 0.74 [0.67-0.80] |
| Age + NIHSS score on admission + MPO concentration | 0.70 [0.64-0.78] |
| Age + neutrophils + MMP-9 concentration | 0.71 [0.65-0.78] |
| Age + neutrophils + MPO concentration | 0.70 [0.63-0.76] |
| Age + MMP-9 concentration + MPO concentration | 0.70 [0.64-0.77] |
| Neutrophils + NIHSS score on admission + MMP-9 concentration | 0.71 [0.65-0.78] |
| Neutrophils + NIHSS score on admission + MPO concentration | 0.70 [0.63-0.77] |
| Neutrophils + MMP-9 concentration + MPO concentration | 0.64 [0.58-0.71] |
| Age + NIHSS score on admission + neutrophils + MMP-9 concentration | 0.75 [0.69-0.81] |
| Age + NIHSS score on admission + neutrophils + MPO concentration | 0.74 [0.68-0.80] |
| Age + NIHSS score on admission + MMP-9 concentration + MPO concentration | 0.74 [0.67-0.80] |
| Age + neutrophils + MMP-9 concentration + MPO concentration | 0.72 [0.65-0.78] |
| NIHSS score on admission + neutrophils + MMP-9 concentration + MPO concentration | 0.71 [0.64-0.78] |

**Tab. S5** Univariable Analysis for Association of Systemic Biomarker Levels and Functional Outcome 3 Months after Stroke and Mortality.

| Variable | Odds ratio  for functional outcome [95% CI] | Odds ratio  for mortality [95% CI] |
| --- | --- | --- |
| Age | 1.04 [1.02-1.06], p=0.0004 | 1.08 [1.05-1.11], p<0.0001 |
| NIHSS on admission | 1.13 [1.08-1.20], p<0.0001 | 1.11 [1.06-1.16], p<0.0001 |
| NIHSS after 24 h | 1.26 [1.19-1.35], p<0.0001 | 1.11 [1.08-1.14], p<0.0001 |
| ASPECT Score | 0.21 [0.11-0.38], p<0.0001 | 0.40 [0.24-0.66], p=0.0004 |
| Recanalization therapy (yes/no) | 0.61 [0.20-1.57], p=0.34 | 0.50 [0.22-1.14], p=0.10 |
| MMP-9 log10 | 15.63 [3.54-80.69], p=0.0005 | 13.93 [4.15-50.21], p<0.0001 |
| MPO log10 | 4.02 [1.84-9.21], p=0.0007 | 4.01 [1.91-8.84], p=0.0004 |

**Tab. S6** Multivariable Analysis for Association of Systemic Biomarker Levels and Functional Outcome after 3 Months with Additional Adjustment for Diabetes Mellitus and pre-stroke mRS.

| Variable | Odds ratio*  [95% CI] | Odds ratio**  [95% CI] | Odds ratio*  [95% CI] | Odds ratio**  [95% CI] |
| --- | --- | --- | --- | --- |
| Age | 1.03 [1.00-1.06] | 1.03 [1.00-1.07] | 1.02 [1.00-1.06] | 1.03 [1.00-1.07] |
| NIHSS score on admission | 1.10 [1.03-1.18] | / | 1.10 [1.03-1.18] | / |
| NIHSS score 24 h after admission | / | 1.22 [1.15-1.33] | / | 1.22 [1.14-1.32] |
| ASPECT Score | 0.17 [0.08-0.35] | 0.34 [0.14-0.77] | 0.16 [0.08-0.34] | 0.32 [0.14-0.74] |
| Recanalization therapy (yes/no) | 0.66 [0.16-2.52] | 0.89 [0.19-4.10] | 0.96 [0.21-3.98] | 1.27 [0.25-6.27] |
| Diabetes mellitus | 2.90 [1.13-8.04] | 2.15 [0.80-6.14] | 3.21 [1.24-9.06] | 2.34 [0.86-6.77] |
| Pre-stroke mRS | 4.33 [2.38-9.67] | 4.45 [2.24-11.06] | 4.49 [2.46-10.12] | 4.80 [2.38-12.18] |
| MMP-9 log10 | / | / | 14.86 [2.51-102.83] | 8.31 [1.10-71.55] |
| MPO log10 | 2.12 [0.88-5.27] | 2.03 [0.72-5.88] | / | / |

**Tab. S7** Multivariable Analysis for Association of Systemic Biomarker Levels and Mortality with Additional Adjustment for Diabetes Mellitus and pre-stroke mRS.

| Variable | Odds ratio*  [95% CI] | Odds ratio**  [95% CI] | Odds ratio*  [95% CI] | Odds ratio**  [95% CI] |
| --- | --- | --- | --- | --- |
| Age | 1.07 [1.04-1.10] | 1.08 [1.04-1.12] | 1.06 [1.03-1.10] | 1.08 [1.04-1.11] |
| NIHSS score on admission | 1.07 [1.02-1.13] | / | 1.07 [1.02-1.12] | / |
| NIHSS score 24 h after admission | / | 1.11 [1.07-1.15] | / | 1.11 [1.07-1.14] |
| ASPECT Score | 0.35 [0.18-0.65] | 0.53 [0.26-1.06] | 0.35 [0.18-0.66] | 0.53 [0.26-1.05] |
| Recanalization therapy (yes/no) | 0.48 [0.15-1.44] | 0.43 [0.13-1.39] | 0.64 [0.21-1.93] | 0.55 [0.17-1.76] |
| Diabetes mellitus | 1.02 [0.48-2.16] | 0.88 [0.37-2.05] | 1.00 [0.45-2.14] | 0.88 [0.37-2.05] |
| Pre-stroke mRS | 1.51 [1.18-1.94] | 1.44 [1.10-1.89] | 1.59 [1.24-2.05] | 1.50 [1.14-1.97] |
| MMP-9 log10 | / | / | 12.53 [2.91-58.34] | 8.92 [1.85-46.14] |
| MPO log10 | 3.32 [1.35-8.65] | 3.34 [1.25-9.49] | / | / |

**Tab. S8** Multivariable Analysis for Association of Systemic Biomarker Levels and 3-month Outcome concerning Patients with Blood Samples taken 24-48 h after Stroke Onset.

| Variable | Odds ratio*  [95% CI] | Odds ratio**  [95% CI] | Odds ratio*  [95% CI] | Odds ratio**  [95% CI] |
| --- | --- | --- | --- | --- |
| Age | 1.05 [1.01-1.09] | 1.04 [1.00-1.09] | 1.04 [1.01-1.08] | 1.04 [1.00-1.08] |
| NIHSS score on admission | 1.09 [1.02-1.18] | / | 1.10 [1.02-1.18] | / |
| NIHSS score 24 h after admission | / | 1.23 [1.13-1.35] | / | 1.23 [1.13-1.35] |
| ASPECT Score | 0.22 [0.08-0.52] | 0.49 [0.16-1.42] | 0.23 [0.09-0.54] | 0.51 [0.17-1.46] |
| Recanalization therapy (yes/no) | 0.36 [0.08-1.42] | 0.43 [0.08-1.89] | 0.54 [0.11-2.31] | 0.63 [0.12-2.93] |
| MMP-9 log10 | / | / | 21.28 [2.36-251.57] | 7.92 [0.64-121.45] |
| MPO log10 | 3.00 [0.89-11.55] | 2.28 [0.53-10.96] | / | / |

Odds ratios are reported for logarithmic increases of base 10.

* adjusted for age, NIHSS score on admission, ASPECTS dichotomized, recanalization therapy.

** adjusted for age, NIHSS score 24 h after admission, ASPECTS dichotomized, recanalization therapy.

**Tab. S9** Multivariable Analysis for Association of Systemic Biomarker Levels and 3-month Outcome concerning Patients with Blood Samples taken ≤ 24 h after Stroke Onset.

| Variable | Odds ratio*  [95% CI] | Odds ratio**  [95% CI] | Odds ratio*  [95% CI] | Odds ratio**  [95% CI] |
| --- | --- | --- | --- | --- |
| Age | 1.05 [1.01-1.10] | 1.08 [1.02-1.15] | 1.06 [1.02-1.11] | 1.08 [1.03-1.15] |
| NIHSS score on admission | 1.09 [1.00-1.20] | / | 1.07 [0.99-1.18] | / |
| NIHSS score 24 h after admission | / | 1.26 [1.14-1.45] | / | 1.25 [1.13-1.43] |
| ASPECT Score | 0.16 [0.05-0.49] | 0.21 [0.05-0.74] | 0.15 [0.04-0.45] | 0.20 [0.05-0.67] |
| Recanalization therapy (yes/no) | 1.87 [0.08-18.42] | 5.01 [0.18-118.17] | 1.75 [0.08-18.17] | 4.59 [0.16-112.41] |
| MMP-9 log10 | / | / | 5.34 [0.36-129.12] | 2.71 [0.15-75.42] |
| MPO log10 | 2.71 [0.82-9.71] | 2.87 [0.72-12.49] | / | / |

Odds ratios are reported for logarithmic increases of base 10.

* adjusted for age, NIHSS score on admission, ASPECTS dichotomized, recanalization therapy.

** adjusted for age, NIHSS score 24 h after admission, ASPECTS dichotomized, recanalization therapy.

**Tab. S10** Multivariable Analysis for Association of Systemic Biomarker Levels and Mortality concerning Patients with Blood Samples taken 24-48 h after Stroke Onset.

| Variable | Odds ratio*  [95% CI] | Odds ratio**  [95% CI] | Odds ratio*  [95% CI] | Odds ratio**  [95% CI] |
| --- | --- | --- | --- | --- |
| Age | 1.09 [1.05-1.13] | 1.10 [1.05-1.15] | 1.08 [1.05-1.13] | 1.10 [1.05-1.15] |
| NIHSS score on admission | 1.08 [1.02-1.15] | / | 1.08 [1.02-1.16] | / |
| NIHSS score 24 h after admission | / | 1.13 [1.09-1.19] | / | 1.13 [1.08-1.19] |
| ASPECT Score | 0.23 [0.10-0.50] | 0.35 [0.14-0.84] | 0.22 [0.09-0.49] | 0.34 [0.13-0.83] |
| Recanalization therapy (yes/no) | 0.40 [0.09-1.59] | 0.32 [0.06-1.47] | 0.53 [0.12-2.19] | 0.39 [0.07-1.86] |
| MMP-9 log10 | / | / | 6.37 [1.04-43.93] | 2.96 [0.40-24.87] |
| MPO log10 | 1.60 [0.52-5.12] | 1.40 [0.38-5.36] | / | / |

Odds ratios are reported for logarithmic increases of base 10.

* adjusted for age, NIHSS score on admission, ASPECTS dichotomized, recanalization therapy.

** adjusted for age, NIHSS score 24 h after admission, ASPECTS dichotomized, recanalization therapy.

**Tab. S11** Multivariable Analysis for Association of Systemic Biomarker Levels and Mortality concerning Patients with Blood Samples taken ≤ 24 h after Stroke Onset.

| Variable | Odds ratio*  [95% CI] | Odds ratio**  [95% CI] | Odds ratio*  [95% CI] | Odds ratio**  [95% CI] |
| --- | --- | --- | --- | --- |
| Age | 1.08 [1.03-1.13] | 1.09 [1.03-1.15] | 1.08 [1.03-1.14] | 1.09 [1.04-1.15] |
| NIHSS score on admission | 1.08 [1.00-1.18] | / | 1.06 [0.98-1.15] | / |
| NIHSS score 24 h after admission | / | 1.10 [1.05-1.16] | / | 1.09 [1.04-1.15] |
| ASPECT Score | 0.81 [0.29-2.26] | 1.17 [0.38-3.70] | 0.89 [0.33-2.43] | 1.25 [0.42-3.87] |
| Recanalization therapy (yes/no) | 0.43 [0.05-3.26] | 0.55 [0.06-4.01] | 0.37 [0.04-2.60] | 0.47 [0.05-3.35] |
| MMP-9 log10 | / | / | 33.38 [2.80-549.67] | 24.41 [2.04-389.18] |
| MPO log10 | 10.06 [2.48-50.96] | 9.20 [2.14-49.37] | / | / |

Odds ratios are reported for logarithmic increases of base 10.

* adjusted for age, NIHSS score on admission, ASPECTS dichotomized, recanalization therapy.

** adjusted for age, NIHSS score 24 h after admission, ASPECTS dichotomized, recanalization therapy.

**Tab. S12** Correlation of Systemic Biomarker Levels with Leukocytes, Neutrophil Granulocytes, Monocytes and Thrombocytes in Blood Sampled over 48 h after Stroke Onset (n=131).

| **Variable** | **systemic plasma levels of biomarker at baseline** | |
| --- | --- | --- |
|  | **p-value** | **r [95% CI]** |
| MMP-9 |  |  |
| differential blood count |  |  |
| Leukocytes | <0.0001 | 0.40 [0.23-0.53] |
| Neutrophil granulocytes | <0.0001 | 0.39 [0.23-0.53] |
| Monocytes | 0.0014 | 0.27 [0.10-0.42] |
| Thrombocytes | 0.0100 | 0.22 [0.04-0.38] |
| Lymphocytes | 0.3506 | 0.08 [-0.10-0.26] |
| Neutrophil-to-lymphocyte ratio | 0.0761 | 0.16 [-0.02-0.32] |
| MPO |  |  |
| differential blood count |  |  |
| Leukocytes | 0.0061 | 0.24 [0.06-0.40] |
| Neutrophil granulocytes | 0.0020 | 0.27 [0.10-0.42] |
| Monocytes | 0.1246 | 0.13 [-0.04-0.30] |
| Thrombocytes | 0.0760 | 0.16 [-0.02-0.32] |
| Lymphocytes | 0.9931 | -0.0008 [-0.18-0.18] |
| Neutrophil-to-lymphocyte ratio | 0.0827 | 0.15 [-0.02-0.32] |

**Fig. S1** Heidelberg Bleeding Classification for Patients with Blood Samples ≤48h according to Biomarker Levels.

Categorization of intracranial bleedings according to the Heidelberg Bleeding Classification (HBC: 1a, hemorrhagic infarction (HI1)—scattered small petechiae; 1b, HI2—confluent petechiae; 1c, parenchymal hematoma (PH1)—hematoma within infarcted tissue, occupying <30%; 2, PH2—hematoma occupying ≥30% of the infarcted tissue; 3a—PH remote from infarcted brain tissue; 3b—intraventricular hemorrhage; 3c—subarachnoid hemorrhage; 3d—subdural hemorrhage). Patients were grouped regarding to bleeding type (HBC classes 1a + b, minor [petechial] intracerebral hemorrhage; HBC classes 1c-3a, major intracerebral hemorrhage [parenchymal hematoma]; HBC classes 3b-d, intracranial-extracerebral hemorrhage); n=273.

**Fig. S2** Prognostic Relevance and Etiological Discrimination Potential of MMP-9 and MPO in Samples over 48 h.

Δ NIHSS (NIHSS score after 24 h - NIHSS score on admission) in patients with biomarker levels according to cut-off value (MMP-9: 298.1 ng/ml; MPO: 25.5 ng/ml). **B)** Biomarker levels and survival after 3 months. **C)** Biomarker levels and atrial fibrillation (AF). **D)** Etiology of stroke for MMP-9 and MPO levels (LAA: large artery atherosclerosis, CE: cardioembolism, ESUS: embolic stroke of undetermined source) (n=127). **E)** ROC analysis of MMP-9 (AUC=0.63, 95% CI: 0.52-0.73) and MPO (AUC=0.60, 95% CI: 0.50-0.71) for functional outcome after 3 months (mRS 0-2 vs. 3-6). *p-value < 0.05; **p-value < 0.01; ***p-value < 0.001; n=138.

**Fig. S3** Spearman’s Correlations for MMP-9 and MPO and **A, B)** Thrombocytes, **C, D)** Lymphocytes, **E, F)** Monocytes within 48 h; n=273.
